# 2,4-Di-tert-butylphenol from Endophytic Fungi *Fusarium oxysporum* attenuates the growth of multidrug-resistant pathogens

**DOI:** 10.3389/fmicb.2025.1575021

**Published:** 2025-06-10

**Authors:** Ranjitha Dhevi V. Sundar, Sathiavelu Arunachalam

**Affiliations:** ^1^Department of Biotechnology, School of Biosciences and Technology, Vellore Institute of Technology, Vellore, India; ^2^Department of Biotechnology, VIT School of Agricultural Innovations and Advanced Learning, Vellore Institute of Technology, Vellore, India

**Keywords:** *Fusarium oxysporum*, global health, antibiotic-resistant, toxicity, chromatography, compound

## Abstract

**Introduction:**

The continuous emergence of drug-resistant pathogenic bacteria highlights the persistent importance of discovering and developing new antibiotics. Endophytic species are an infinite source of several medicinally essential secondary metabolites.

**Methods:**

The current study focuses on isolating secondary metabolites from the endophytic *Fusarium oxysporum* TPL11 fungus of *Tradescantia pallida* plant. These fungi were spectrally analyzed by nuclear magnetic resonance (NMR), Fourier transform infrared spectroscopy (FTIR), gas chromatography-mass spectrometry (GC-MS), and the structures were predicted. The pure compounds were tested for antagonistic susceptibility to multidrug-resistant (MDR) pathogens using disk diffusion, minimum inhibitory concentration (MIC), minimum bactericidal concentration (MBC), time-kill kinetics, and cytotoxicity assays.

**Results and discussion:**

Bioactivity-guided extraction of ethyl acetate fungal extract purification yielded a lead compound, 2,4-di-tert-butylphenol (DTB), which was interpreted by combining ^1^H NMR, ^13^C NMR, and mass spectrometry (MS) data. The compound DTB displayed antagonism against human pathogens with diameters ranging from 16 to 26 mm. The highest antagonistic effect was against methicillin-resistant *Staphylococcus aureus* (MRSA) (ATCC 700699) and VRE (ATCC 51299) with 21 ± 0.2 and 22 ± 0.5 mm zones of inhibition, respectively. The compound had MICs of 3.12 and 6.25 μg/mL, and MBCs of 0.78 and 3.12 μg/mL against MRSA (ATCC 700699) and vancomycin-resistant *Enterococcus faecalis* (VRE) (ATCC 51299), respectively. The time-kill kinetics study reveals that DTB displayed a bactericidal effect against pathogens at higher concentrations, indicating a concentration and time-dependent interaction. In a cytotoxicity assay, it is more active against the cell line with a decrease in cell viability of 50.12% at 1,000 μg/mL concentration. The results highlighted that compound DTB from *F. oxysporum* showed significant pharmaceutical potential, indicating its suitability as a lead molecule. The study outcome suggests that the active lead metabolites isolated for the first time from *F. oxysporum* isolated from *T. pallida* plant can be an auspicious antibacterial agent for controlling multidrug-resistant pathogens marking a novel discovery in this domain.

## Graphical Abstract

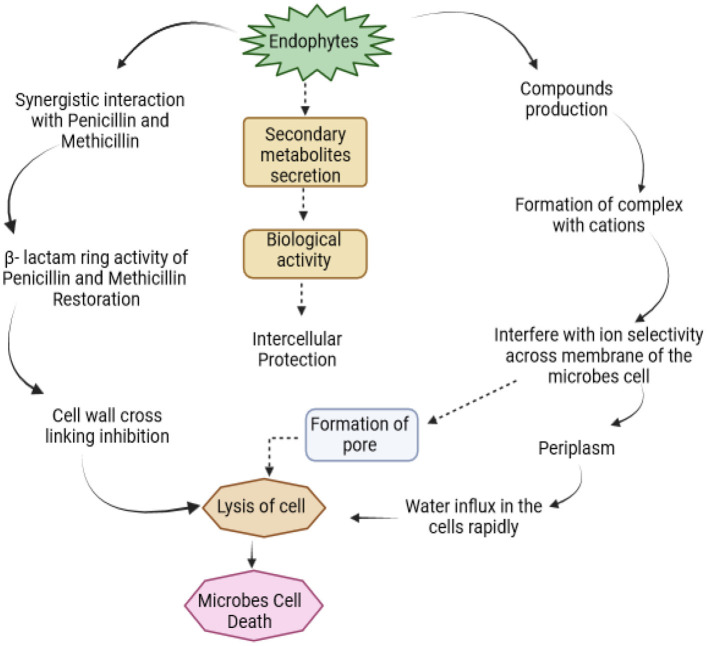

## 1 Introduction

Antibiotic resistance, often referred to as superbugs, is the ability of pathogens or other microbes to survive and reproduce in the presence of previously effective antibiotic dosages (Mule et al., 2020). This resistance occurs spontaneously when microorganisms come into contact with antibiotic drugs. Antibiotics are intended to eradicate or suppress susceptible bacteria selectively; nevertheless, microorganisms with inherent or acquired drug-resistant features are more likely to survive and proliferate (Prestinaci et al., 2015). When antibiotics are used inappropriately or unnecessarily, bacteria can adapt and evolve, resulting in the growth of resistant strains (Abdulmyanova et al., 2020). Excessive usage of antibiotics has contributed to an upsurge in infections due to antibiotic-resistant microbes, notably multiple drug-resistant strains. A growing number of infectious microbes that do not respond to medications poses a threat to the global healthcare system (Janeš et al., 2007). Antimicrobial resistance (AMR) is not unusual; however, the current situation is marked by an unprecedented and continuous increase in resistant microbes, vast geographical zones affected by antibiotic resistance, and a growing range of resistance within distinct organisms (Levy and Marshall, 2004). Infections stemming from antibiotic-resistant *S. aureus* pose a substantial menace to global public health. The most prevalent Gram-positive pathogens contributing to this threat include methicillin-resistant *Staphylococcus aureus* (MRSA), antibiotic-resistant *Streptococcus pneumoniae*, multidrug-resistant (MDR) *Mycobacterium tuberculosis*, and VRE strain (Ratnaweera et al., 2018). *Staphylococci* have been the cause of a range of human illnesses, encompassing circumstances including prolonged wound ailments, periodontitis, osteomyelitis, endocarditis, and infections related to implanted medical devices (Sadrati et al., 2023).

In the field of ethnobotany concerning the safeguarding, preservation, and sustainable utilization of plants, researchers have focused on the extraction and study of microorganisms associated with plant life (Verma et al., 2022). Plants serve as a considerable reservoir for a wide variety of microbes known as endophytes (Sangeetha et al., 2020). Endophytes are microbes that live within the endosphere of plants without causing any infectious signs, triggering evident morphological changes in their host plant or organism (Mishra and Priyanka Sharma, 2022). Several reports from past decades showed that ethnomedicinal plant species rich in botanical history were analyzed from exclusive species of ecological niches that were known to possess effective endophyte microbes (Nalini et al., 2014). The majority of isolated endophytes are fungi, presenting significant prospects for discovering unfamiliar fungal species, given that a substantial portion of plant-associated endophytes remains unexplored (Strobel and Daisy, 2003). A wide range of biological activities of endophytic fungi has been reported from different plants (Patle et al., 2018).

In our previous study, 14 (from TPL1 to TPL14) endophytic fungi were isolated from the leaves of *Tradescantia pallida* (Rose) D. R. Hunt var. *purpurea*. They were screened for antibacterial activity, of which the ethyl acetate crude extract of *Fusarium oxysporum* TPL11 showed maximum antagonism toward the tested bacterial pathogens (Sundar and Arunachalam, 2024). In the present study, the most active *F. oxysporum* TPL11 obtained from the previous study was mass cultured to extract and purify potent antibacterial secondary metabolites through a bioassay-guided fractionation procedure.

## 2 Materials and methods

### 2.1 General experimental procedures

This study employed comprehensive analytical techniques to investigate the molecular composition and structure of the target compound. Fourier transform infrared (FTIR) spectroscopy was performed using potassium bromide (KBr) pellets on a Shimadzu FTIR 8300 instrument over the 4,000–400 cm^−1^ spectral range. The FTIR analysis provided valuable information about the functional groups present in the sample. A Micromass ZQTM 4000 mass spectrometer equipped with Electrospray Ionization Mass Spectrometry (ESI-MS) was employed for mass spectrometric analysis. This technique allowed for determining the molecular weight and identifying the ions produced by the sample. Additionally, nuclear magnetic resonance (NMR) spectroscopy was conducted using a Bruker Advance 400 NMR spectrometer operating at 400 MHz for ^1^H and 100 MHz for ^13^C nuclei. To further purify and isolate the compound of interest, silica gel chromatography was carried out using silica gel with a mesh size of 60–120 from Hi-Media.

### 2.2 Solvent system optimization

The ethyl acetate (EA) crude extract exhibited maximum activity against all the bacterial pathogens tested. TLC was performed at room temperature in a TLC chamber to analyze the polarity of the compounds in the fungal extract. The crude extracts were dissolved in ethyl acetate solvent, and spots were placed on a TLC plate (6 × 2 cm), which was then placed inside the chamber containing solvents of Chloroform: EA: Methanol (6:4:3) ratio. The TLC was run through experimentation with different organic solvent combinations, such as 7:5:4, 8:6:5, 9:7:6, 5:3:2, and 4:2:1. The ratio that shows distinct band separation was used as the optimal ratio for column chromatography (Sharma et al., 2016).

### 2.3 Detection of crude antagonism by TLC-contact bioautography

TLC-contact bioautography testing was performed to assess the antagonistic ability of bioactive constituents of fungal-extracted substances against human pathogens. The bacterial strains, such as. Methicillin-resistant strains of *S. aureus*—MRSA (ATCC 43300, ATCC 700699), *S. aureus* (ATCC 25923, MTCC 3160), Vancomycin-resistant enterococci (VRE) (ATCC 51299), and *E. faecalis* were used for the study. The pathogens were freshly cultured in Tryptic Soy Broth (TSB) and Brain Heart Infusion (BHI) broth. The bacterial suspensions were then standardized to 0.5 McFarland, corresponding to a concentration of 107−108 CFU/mL, and maintained at −4°C, and then swabbed onto the freshly prepared MHA dishes. The crude extract was spotted on the TLC and run with the desired solvent ratio. Then, developed chromatogram (TLC) plates comprising separated compounds of the extracts were dried. Then it is placed facing down over the medium aseptically for 30 min to 1 h to assist the diffusion of bioactive substances. The inoculated agar plate was sealed with paraffin and incubated for 24 h at room temperature (37°C). The inhibitory zone on the agar surface that correlates to the TLC spots demonstrates that the active constituents in the fungal crude have antibacterial potency (Astuti et al., 2014).

### 2.4 Mass production of active endophytic fungus

The fungus *F. oxysporum* (TPL11) crude extract that showed the highest activity and maximum yield was chosen for large-scale fermentation. Inocula (agar plugs) of active isolates were introduced to 1 L flasks (5–10) containing potato dextrose broth (PDB) and subjected to submerged fermentation at 27°C for 21 days. The filtered broth after fermentation was filtered using Whatman filter paper (No. 1) to separate broth and the mycelium, and extracted with an equal volume of ethyl acetate solvent using a separating funnel and concentrated using a rotatory evaporator to give crude extract (Kaaniche et al., 2019; Goutam et al., 2016).

### 2.5 Purification of bioactive metabolites by silica gel column chromatography

To further purify the active component in the EA crude extract, silica gel column chromatography with a mesh size of 60–120 was used as the stationary phase. To prepare, a slurry was made by mixing 3 g of the EA crude extract with silica gel. The mobile phase comprises an optimized solvent ratio [chloroform:EA:methanol (6:4:3)]. Fractions were collected at a constant flow rate of 1 mL/min, with a flow volume of 20 mL for each fraction. TLC was next performed, and fractions with comparable patterns based on their retention factor were merged and concentrated. The antibacterial efficacy of all pooled fractions against MDR human pathogens was then evaluated. The purified compound underwent further identification studies (Kaaniche et al., 2019; Goutam et al., 2016).

### 2.6 Structural elucidation of the pure compound

#### 2.6.1 Identification of bioactive compounds through gas chromatography-mass spectrometry

Gas chromatography (GC) and mass spectroscopy separated and identified the pure compound. The analysis used a Perkin Elmer Clarus 680 instrument attached to the Mass spectrum component. The initial temperature of the oven was maintained at 60°C for 2 min, ramp (10 min) to 300°C, hold 6 min. The temperature of the injector was 25°C. The carrier gas used here was helium with a constant flow rate of 1 mL/min. At 240°C, the mass transfer line and source temperature were set. Spectrum was analyzed by Turbo Mass version 5.4.2 software, and the structure was determined by mass spectral patterns to the National Institute of Standards and Technology (NIST) library (2008) comparison (Kanjana et al., 2019).

#### 2.6.2 Fourier transform infrared spectroscopy characterization of isolated compound

FTIR analysis was performed for the dried isolated compound using a Shimadzu FTIR spectrophotometer (model: IR Affinity). The chemical compound functional groups present in the compound were recorded in the 4,000–400 cm^−1^ range, and the spectrum was attained under 4 cm^−1^ resolution. The infrared absorption spectrum is used to determine the chemical bonds in the molecule. The annotated spectrum specifies that the chemical bonds present in the sample absorb a specific wavelength of light (Kanjana et al., 2019).

#### 2.6.3 NMR characterization

The pure compound NMR spectral analysis was performed on an NMR spectrometer to identify the chemical shifts of carbon, proton atoms in the purified compound. The solvent used for NMR analysis was deuterated chloroform (CDCl3). About 5 mg of the pure compound was dissolved in 400 mL of CDCl_3_. Topsin software was used to visualize and plot the NMR results obtained. The integration of these analytical techniques, including FTIR spectroscopy, ESI-MS, NMR spectroscopy, and silica gel chromatography, allowed for a thorough characterization of the chemical properties of the investigated compound, contributing to a comprehensive understanding of its molecular structure and composition (Kaliaperumal et al., 2023).

### 2.7 Antagonistic activity by the disk diffusion method

The isolated pure compound obtained from the ethyl acetate crude extract was solubilized in dimethyl sulfoxide (DMSO) at a concentration of 5% to determine the antibacterial activity by the disk diffusion procedure. Subsequently, the prepared sample was carefully applied to a sterile disk, which was then positioned onto a culture medium pre-inoculated with the corresponding bacterial strain. Following the application of the sample, the Petri dishes were incubated at a temperature of 37°C for a duration of 12–18 h. To quantify the inhibitory effect, the diameters of these clear zones were measured. Three independent technical replicates were conducted to ensure the reliability of the results (Samapti et al., 2022).

#### 2.7.1 Antibacterial activity index

The activity index (AI) for each isolated compound was calculated by following the formula (Singh and Kumar, 2012):


$$\begin{array}{r} \text{ AI }=\text{ Zone of inhibition }\left(\text{ZOI}\right)\text{ of sample}/\text{Inhibition zone}\\ \text{of inhibition of standard} \end{array}$$

### 2.8 MIC and MBC of the pure compound

Determining minimum inhibitory concentrations (MICs) for fungus extracts was primarily conducted through a microdilution method employing 500 μL Muller–Hinton broth. The MIC was determined using a serial dilution approach implemented in 96-well microtiter plates. Each well was loaded with 50–0.78 μg/mL of extract, followed by the addition of 10 μL of bacterial cell suspensions. Subsequently, the microplates underwent a 12–18 h incubation period at 37°C. The concentrations exhibiting no visible bacterial growth were identified as the MICs, indicating the minimum concentration required to inhibit bacterial growth entirely. Muller–Hinton broth functioned as the negative control. Minimum bactericidal concentration (MBC) was calculated after reading the MIC titer plate results by transferring a 50 μL mixture from two dilution levels higher and lower than the value of MIC on the MHA plates. The pathogen inoculant was applied onto the medium using a sterile heat-flamed L-rod and incubated for 24 h at 37°C. The MBC of fungal crude extract is defined as not having any bacterial pathogen colonies on the surface of the agar at the lowest concentration of a sample. Positive controls for MRSA and VRE included Oxacillin and Vancomycin (Valle et al., 2016).

### 2.9 Time-kill kinetics study

A time curve analysis of the extract and compound was performed based on the MIC values. The crude extract was meticulously prepared at concentrations equivalent to half, one, and two times the MIC value (1/2MIC, 1MIC, 2MIC). These concentrations were employed to investigate the extract's impact on bacterial growth. A volume of 1 mL from each precisely prepared extract concentration was introduced into conical flasks, each containing 18.9 mL of Mueller-Hinton Broth (MHB). Subsequently, 0.1 mL of a bacterial suspension with a density of 1 × 10^8^ CFU/mL was inoculated into each flask. The cultures were then exposed to a period of incubation at 37°C for 48 h in an orbital shaking incubator with an aeration and agitation speed of 150 rpm. At designated time intervals (0, 4, 8, 12, 16, 20, 24, 28, 32, 36, 40, 44, and 48 h), 0.1 mL aliquots were withdrawn from each culture for viable cell count examination. For each extract concentration, including the control, a time-kill study curve was constructed, displaying log CFU/mL vs. time. The equation below was used to compute the growth decrease, which represents the interval necessary to accomplish 50, 90, and 99.9% reduction in cells of bacteria. It is essential to note that the entire trial was done in triplicate to confirm data reliability and repeatability (Muazzam and Darah, 2021).


$$\begin{array}{l} \%\text{Growth reduction }=\text{vi}-\text{vz}/\text{vz }\times \;100 \end{array}$$

where v_i_ is the initial viable cell count and v_z_ is the viable cell count at time z.

### 2.10 *In silico* docking analysis: ligand selection and preparation

According to the GC-MS investigation, metabolites from potent fungi were selected as ligands. Canonical Simplified Molecular Input Line Entry Specification (SMILES) of each ligand was acquired from PubChem (pubchem.ncbi.nlm.nih.gov) and submitted to the framework CORINA online software (http://www.molecular-networks.com/products/corina). It uses SMILES as input and generates a ligand three-dimensional (3D) structure, which can be downloaded in PDB format and processed to the Protein Data Bank (PDB), Partial Charge (Q), and Atom Type (T) (PDBQT) necessary for AutoDock Vina.

### 2.11 Molecular docking studies

#### 2.11.1 Protein preparation

Structure-activity relationship-based computational docking analysis enhances the understanding of drug–target interactions. The docking studies were implemented with AutoDock Vina (4.2.6) to study the interaction between the pure compound and the drug–target proteins of pathogenic bacteria. The antibacterial targeted proteins' 3D structures were acquired from the Research Collaboratory for Structural Bioinformatics (RCSB) protein data bank (PDB) (http://www.rcsb.org/pdb/home/home.do) (Table 1). The proteins are converted to PDBQT format for docking simulations after removing the hetero atoms and water and adding the polar hydrogen group to all protein structures using the automated docking tool AutoDock Vina. This step ensured a focused analysis of the interaction between the antibacterial compound and the essential amino acid components of the drug–target proteins (Mir et al., 2022; Prajapati et al., 2023).

**Table 1:** List of targeted proteins of bacteria with PDB-ID for docking studies.

**S.No**	**Protein name**	**PDB-ID**
1	Penicillin-binding protein 2a	1VQQ
2	Dehydrosqualene synthase Y129A	3LGZ
3	FtsA	3WQU

#### 2.11.2 Protein-ligand interaction and visualization

AutoDock Vina was employed to perform molecular docking analyses, where ligands docked individually to each receptor using specific grid coordinates. The grid box size for X, Y, and Z dimensions was set to 20 × 20 × 20; the default exhaustiveness of 8 remained during the docking algorithm execution. The protein-ligand binding affinities are determined by evaluating the negative scores of Gibbs free energy in kcal/mol, as computed by AutoDock Vina's scoring feature. The software generated nine poses for each ligand. Subsequently, in BIOVIA Discovery Studio software, the results were analyzed, and the conformation with the best scoring, indicating the lowest binding energy in kcal mol^−1^ and a higher number of hydrogen bonds, was identified. This information was then visualized for further interpretation (Mir et al., 2022; Prajapati et al., 2023).

### 2.12 *In vitro* toxicity analysis on vero cell line

The vero cell lines were sourced from the National Centre for Cell Sciences (NCCS), Pune. Dulbecco's Modified Eagle Medium (DMEM) enriched with fetal bovine serum (FBS) (10%), penicillin (100 U/mL), and streptomycin (100 μg/mL), within a controlled environment at 37°C with 5% CO_2_ in a humidified condition, in which the cells were propagated.

#### 2.12.1 3-(4,5-Dimethyl-2-thiazolyl)-2,5-(diphenyl-tetrazolium bromide) assay (MTT)

A concentration of 1 × 10^5^ cells was dispensed in 24-well microplates and maintained at room temperature in an environment comprising CO_2_ (5%). Then, the cells were exposed to varying concentrations (7.8–1,000 μg/mL) of the test samples for 24 h. After the incubation period, the samples were aspirated, and then the cells were carefully washed with either phosphate-buffered saline (PBS), pH 7.4, or serum-free DMEM. A solution of 0.5% (MTT) at a volume of 100 μL per well (concentration: 5 mg/mL) was added and allowed to incubate for 4 h. Then, 1 mL of dimethyl sulfoxide (DMSO) was introduced into individual wells to dissolve the formazan crystals. OD was then estimated at 570 nm wavelength with a ultraviolet-visible (UV-Vis) spectrophotometer, with DMSO as the reference. The data obtained were analyzed graphically to ascertain the concentration at which 50% inhibition (IC_50_) was observed (Kalimuthu et al., 2022). The percentage of cell viability was determined using the formula:


$$\begin{array}{r} \%\;Cell\;viability\;=\;A570\;of\;treated\;cells/A570\;of\;control\;cells\\ \times 100\; \end{array}$$

### 2.13 Data statistical analysis

The data from triplicate assays were analyzed, and the standard deviation (SD) was calculated for each set of experimental results. Statistical significance was determined by calculating a *p*-value of ≤ 0.05 through one-way ANOVA. The analysis was conducted using version 9.5.1 (733) GraphPad Prism software.

## 3 Results

### 3.1 Selection of fungus for mass production of secondary metabolite extraction

Based on potential bioactivity, the endophyte strain *F. oxysporum* (TPL11) showed maximum inhibitory effect against the resistant human pathogens and was selected for further secondary metabolite extraction using its ethyl acetate extract. Large-scale (10 L) culture and preparation of extract were implemented in a PDB liquid medium with the above method mentioned. Post-fermentation, the broth was extracted with solvent (ethyl acetate), dried, and then the crude extract was subjected to secondary metabolite extraction by column chromatography.

### 3.2 Purification of the compound from *F. oxysporum* (TPL11)

**Figure 1:**
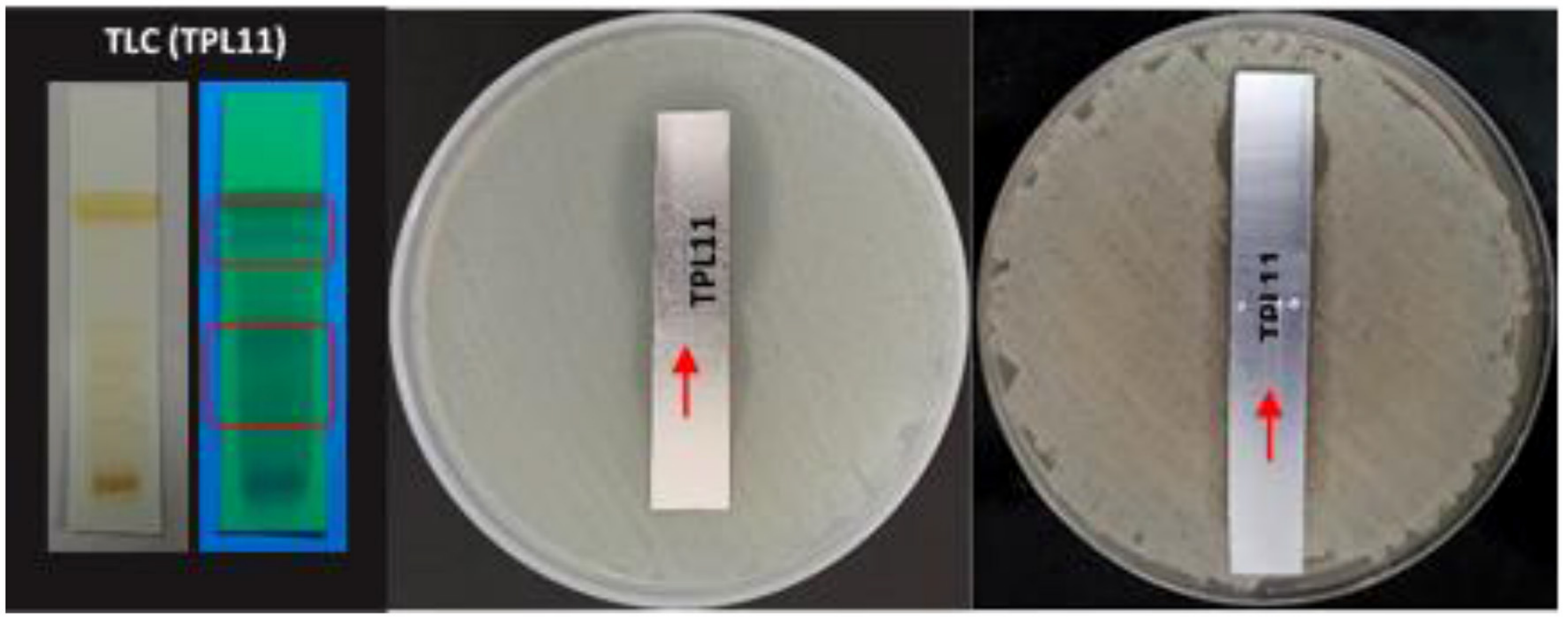
TLC bioautography profile of the *Fusarium oxysporum* (TPL11) ethyl acetate crude sample.

**Figure 2:**
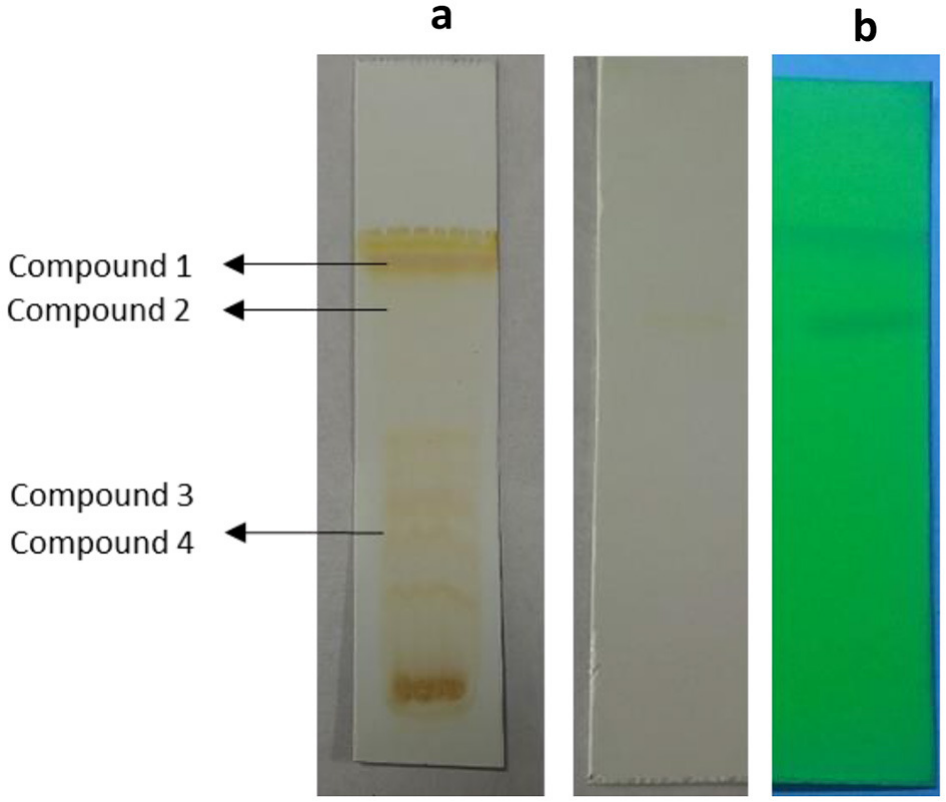
**(a)** TLC profile of *Fusarium oxysporum* (TPL11) ethylacetate crude extracts and **(b)** separated pure compounds.

TLC bioautography was performed to classify the compounds responsible for antibacterial activity (Figure 1). The major compounds responsible for antibacterial activity within the extract showed a ZOI around the separated compounds. Approximately 3 g of EA extract was obtained and subjected to separation through silica gel column chromatography, employing a solvent system for TPL11 composed of chloroform: EA: Methanol (6:4:3) as predetermined by TLC (Figure 2). This process resulted in the isolation of 50 fractions, which were then pooled based on similar retention factors (fractions of 12), concentrated, and evaluated for their antibacterial efficacy.

The active fraction underwent purity analysis by TLC, revealing four pure compounds from TPL11. Among pure compounds with an Rf value of 0.88 (TPL11–L11C1), the highest antibacterial activity against the human pathogens was observed, and they were analyzed for their MIC and time-kill kinetics. Notably, the TPL11–L11C1 compound presented substantial antibacterial activity against resistant bacterial pathogens, thereby subjecting it to structure elucidation.

### 3.3 Spectroscopic data of the pure compound from *F. oxysporum* (TPL11)

**Figure 3:**
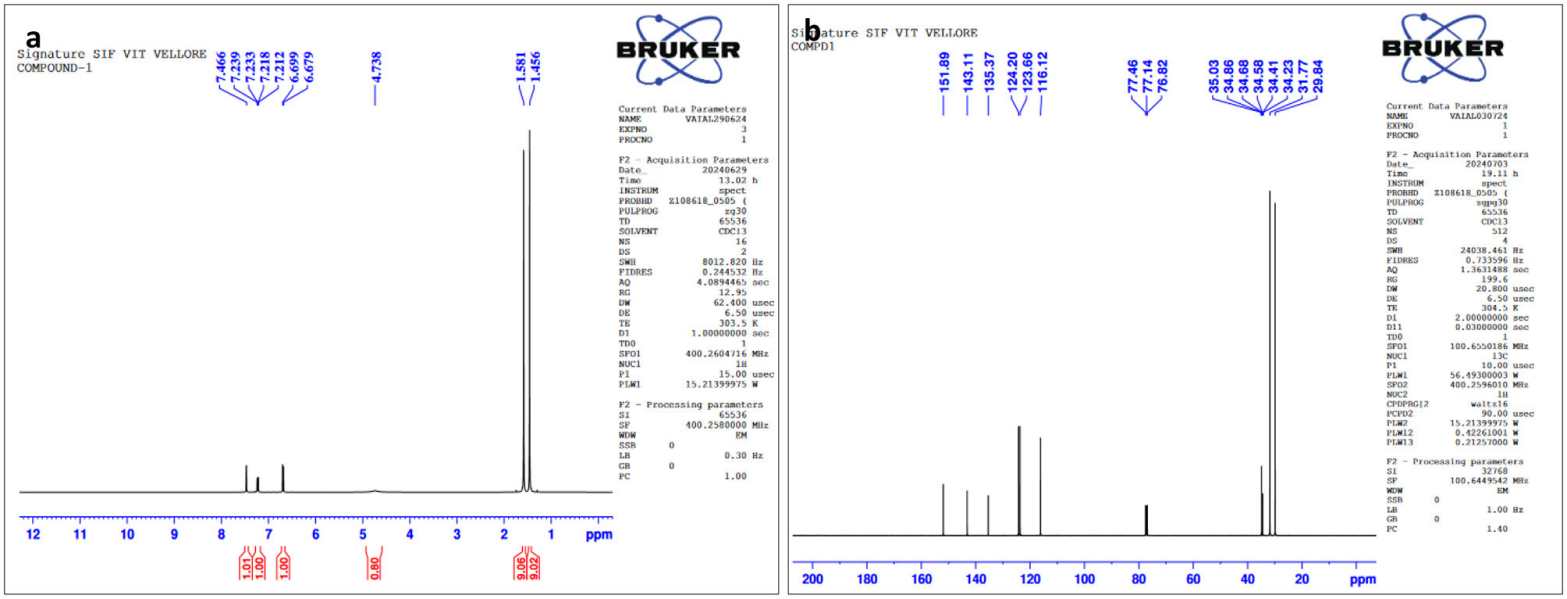
**(a)** ^1^H NMR and **(b)** ^13^C NMR spectral assessment of L11C1 compound isolated from *Fusarium oxysporum*.

**Figure 4:**
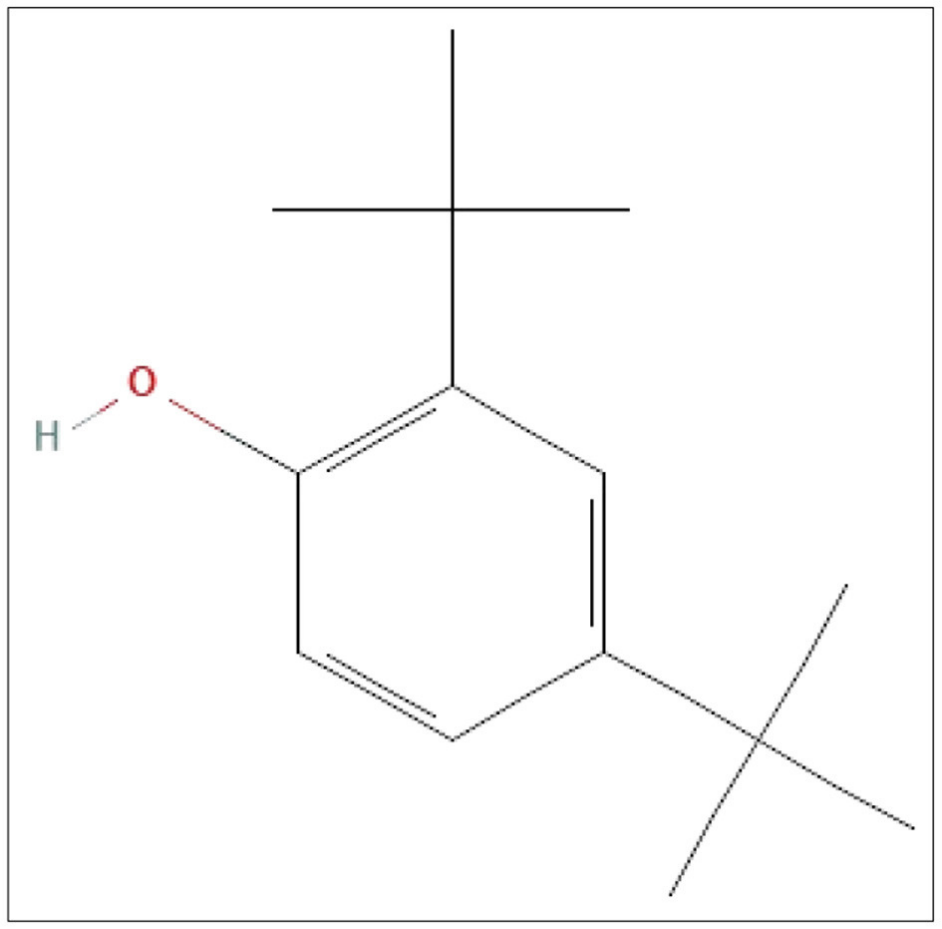
Chemical structure of the isolated pure compound 2,4-di-tert-butylphenol (DTB).

**Figure 5:**
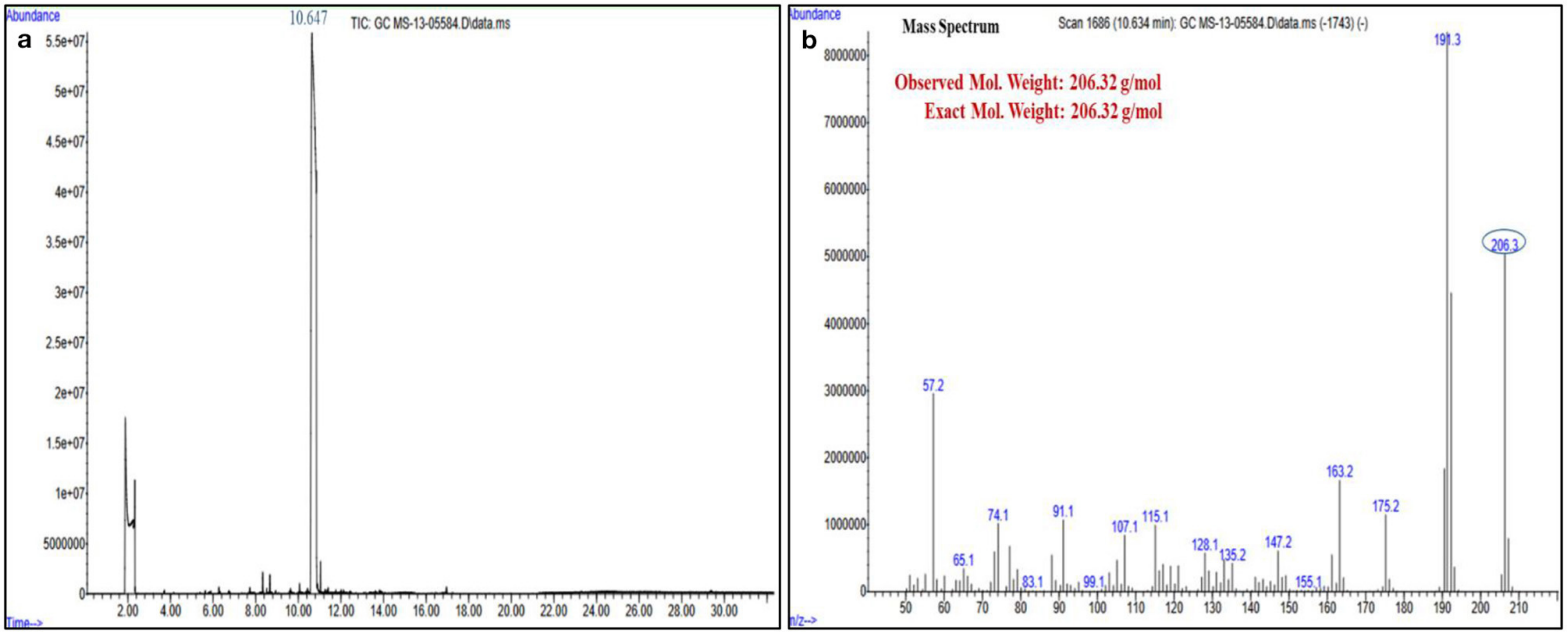
**(a)** Gas Chromatography-Mass Spectrometry and **(b)** Mass spectrum analysis of the active pure fraction (L11C1) of *Fusarium oxysporum*.

The structures of this compound were interpreted through spectral investigation by GC-MS, NMR, and FTIR. The ^13^C NMR (100 MHz, CDCl_3_) study of the compound revealed 10 distinct phase shifts. The recorded peaks were observed at the following chemical shifts: 151.89, 143.11, 135.37, 124.20, 123.66, 116.12, 77.46, 77.14, 76.82, 35.03, 34.86, 34.68, 34.58, 34.41, 34.23, 31.77, and 29.84 ppm, respectively. The compound's ^1^H NMR (400 MHz, CDCl_3_) spectrum showed a sharp peak at 1.456 and 1.581 ppm. The pure compound's ^1^H and ^13^C NMR spectra are presented in Figures 3, 4. The GC-MS analysis exposed a single sharp peak at the retention time of 10.647. Mass spectrum analysis of the purified compound revealed a molecular mass of 206.32 Da. The fragmentation pattern of the interpreted structure corresponds with the spectral data shown in Figure 5. The ^1^H, ^13^C NMR and MS data of L11C1 corresponded to 2,4-di-tert-butylphenol (DTB). 2,4-Di-tert-butylphenol is a substituted phenol, and its phenolic nature is significantly influenced by the bulky tert-butyl groups at the 2- and 4-positions on the aromatic ring.

**Figure 6:**
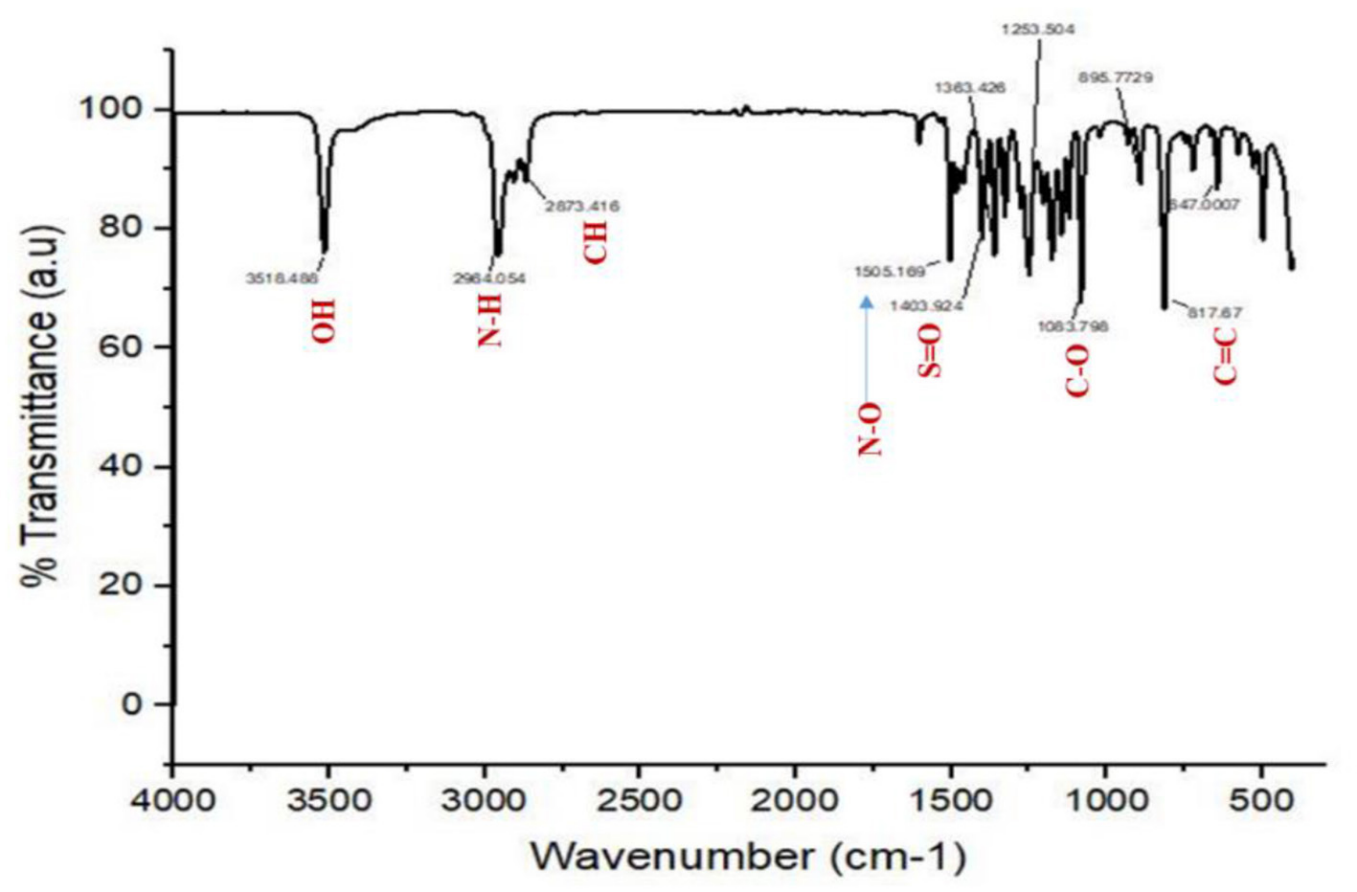
FTIR spectrum of the active pure compound (L11C1) derived from *F. oxysporum* (TPL11).

The FTIR spectrum of the pure compound 1 revealed the existence of distinct functional groups. Specifically, the peaks observed at 3,518.488 cm^−1^ corresponded to the O–H group, while the peak at 1,363.426 cm^−1^ indicated the O–H phenol group. Additionally, the peak at 2,964.054 cm^−1^ suggested the existence of an amine salt group, while 2,873.416 and 1,505.169 cm^−1^ relate to the alkane and N–O groups. The peak at 1403 cm^−1^ may be due to the existence of the sulfate group, while the peaks at 1,253.504, 1,083.795, and 895.77 cm^−1^ are due to the amine, C–O, and alkene groups. The IR spectrum of L11C1, positioning the different functional groups, is depicted in Figure 6.

### 3.4 Antagonistic efficacy of the compound

**Figure 7:**
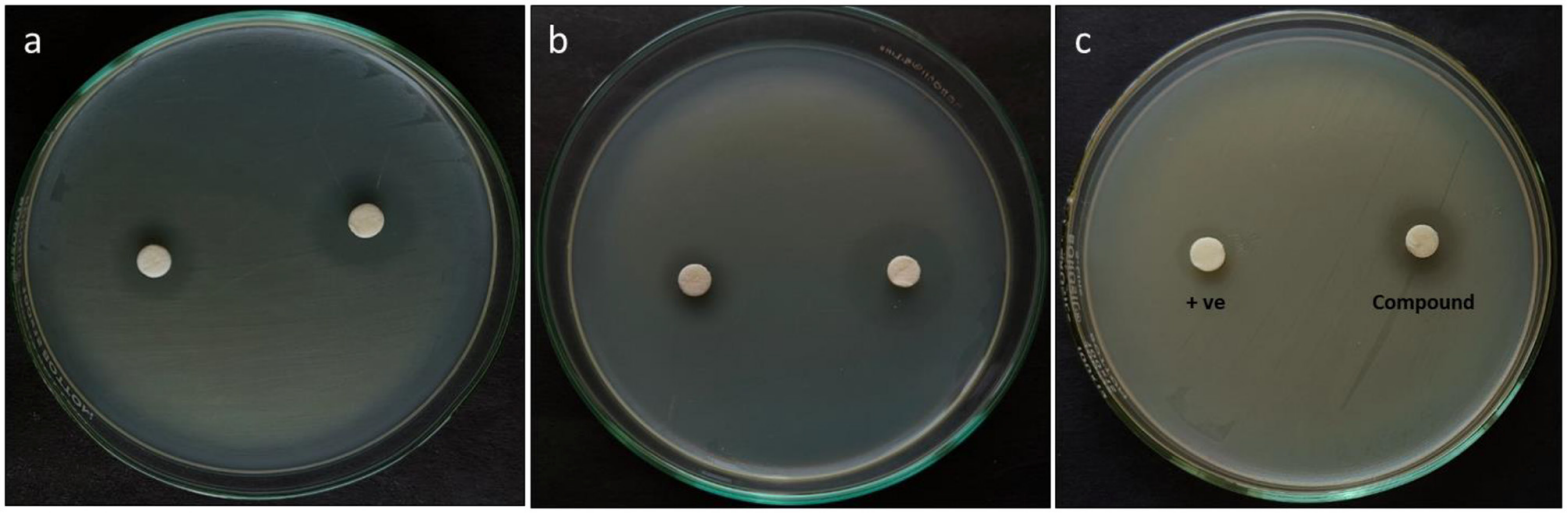
Antibacterial activity of the DTB compound against the MDR pathogens **(a)** MRSA (ATCC 43300), **(b)** MRSA (ATCC 700699), and **(c)** VRE (ATCC 51299).

The antagonistic effectiveness of L11C1 (DTB) was determined by the disk diffusion assay, MIC, MBC, and time-kill kinetic analysis. DTB exhibited a substantial ZOI against all tested resistant pathogens, as illustrated in Figure 7. Among the bacterial strains examined, DTB revealed the maximum antagonistic activity against MRSA (ATCC 700699), a ZOI of 21 mm; AI of 1.4 with MIC of 3.12 and MBC of 0.78 μg/mL, whereas against MRSA (ATCC 43300), with a ZOI of 19 mm; AI of 1.2 with MIC of 3.12 and MBC of 0.78 μg/mL. Moreover, VRE displayed susceptibility, evidenced by a ZOI value of 16 mm with an MIC of 6.25 and an MBC of 3.12 μg/mL. Whereas, the positive control Oxacillin and Vancomycin commercial antibiotics showed MIC of 25 μg/mL and MBC of 50 μg/mL, which was two to 3-fold reduced activity compared to that of the DTB, suggesting that the compound is highly potent toward the test pathogens. The comprehensive results of this investigation are summarized in Table 2.

**Table 2:** Antibacterial activity of the DTB compound against resistant human pathogens.

**Pathogens**	**Concentration (**μ**g/mL)**				
	**DTB**				
	**ZOI**	**AI**	**MIC**	**MBC**	**MIC Index**
MRSA (ATCC 43300)	19 ± 0.05	1.2	3.125	0.78	0.24
MRSA (ATCC 700699)	21 ± 0.2	1.4	3.125	0.78	0.24
*S. aureus* (ATCC 25923)	25 ± 0.24	1.6	0.39	0.39	1
*S. aureus* (MTCC 3160)	26 ± 0.11	1.7	0.78	0.39	0.5
VRE (ATCC 51299)	16 ± 0.2	1	6.25	3.125	0.5
*E*. *faecalis* (ATCC 29212)	22 ± 0.05	1.4	0.78	0.39	0.5
Oxacillin	15 ± 0.12	1	25	50	2
Vancomycin	16 ± 0.24	1	25	50	2

Values expressed as ZOI (mm) ± SD of crude extract (*n* = 3). Positive control: Oxacillin (1 mcg/disk); Negative control: DMSO (5%).

### 3.5 Time-kill kinetics test

**Figure 8:**
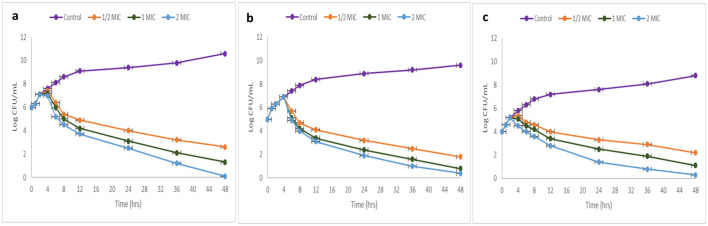
Time-kill kinetics curve of DTB compound **(a)** MRSA (ATCC 43300); **(b)** MRSA (ATCC 700699); and **(c)** VRE.

The kinetics investigation examined the effect of TPL11 (L11C1) on MRSA and VRE over 48 h, testing various concentrations (1/2 MIC, 1 MIC, and 2 MIC). The outcomes indicate that the compounds displayed a bactericidal effect against pathogens at higher concentrations, indicating a concentration and time-dependent interaction. A logarithmic curve of CFU/mL over time was plotted (Figure 8).

### 3.6 Docking result of DTB with bacterial protein

To predict whether the DTB possesses antibacterial activity, molecular docking studies were carried out. AutoDock Vina was used for the interactions, and the results were visualized using BIOVIA Discovery Studio. Bacterial target proteins that were reported previously were chosen from the database.

**Figure 9:**
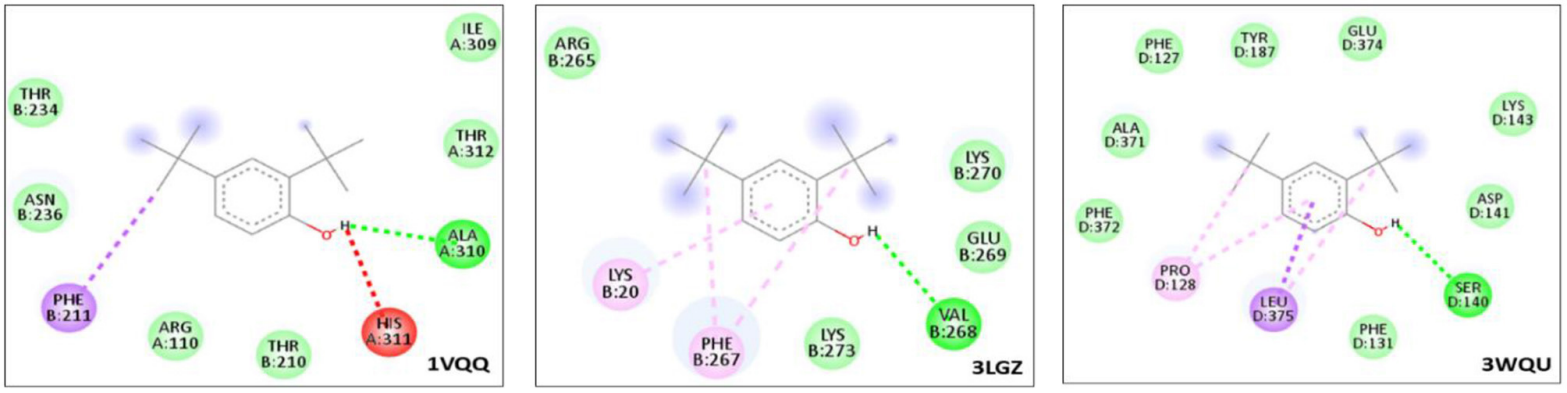
Graphical illustration of protein-ligand docking 2D interaction of DTB with target bacterial proteins.

Among the three proteins (PDB-ID: 1VQQ, 3LGZ, and 3WQU) analyzed, DTB revealed the highest affinity to the 3WQU protein, with a binding energy of −6.7 Kcal/mol. DTB exhibited a strong hydrophobic interaction and formed 9 hydrogen bonds with PHE-127, TYR-187, GLU-374, LYS-143, ASP-141, SER-140, PHE-131, LEU-375, PRO-128, PHE-372, and ALA-371, as illustrated in two-dimensional (2D) views in Figure 9.

Based on these *in silico* molecular docking data, it appears that DTB inhibits 3WQU (FtsA) to mediate its antibacterial effect, indicating strong stability and affinity between the antibacterial metabolites and the protein targets (Table 3).

**Table 3:** Interactions formed by DTB docked with targeted bacterial proteins.

**Proteins (PDB-ID)**	**Binding energy kcal/mol**	**Number of bonds: Hydrogen bond interaction**	**Hydrophobic interactions**
1VQQ	−6.3	7	1
3LGZ	−6.0	5	2
3WQU	−6.7	9	2

### 3.7 *In vitro* toxicity analysis on vero cell line

**Figure 10:**
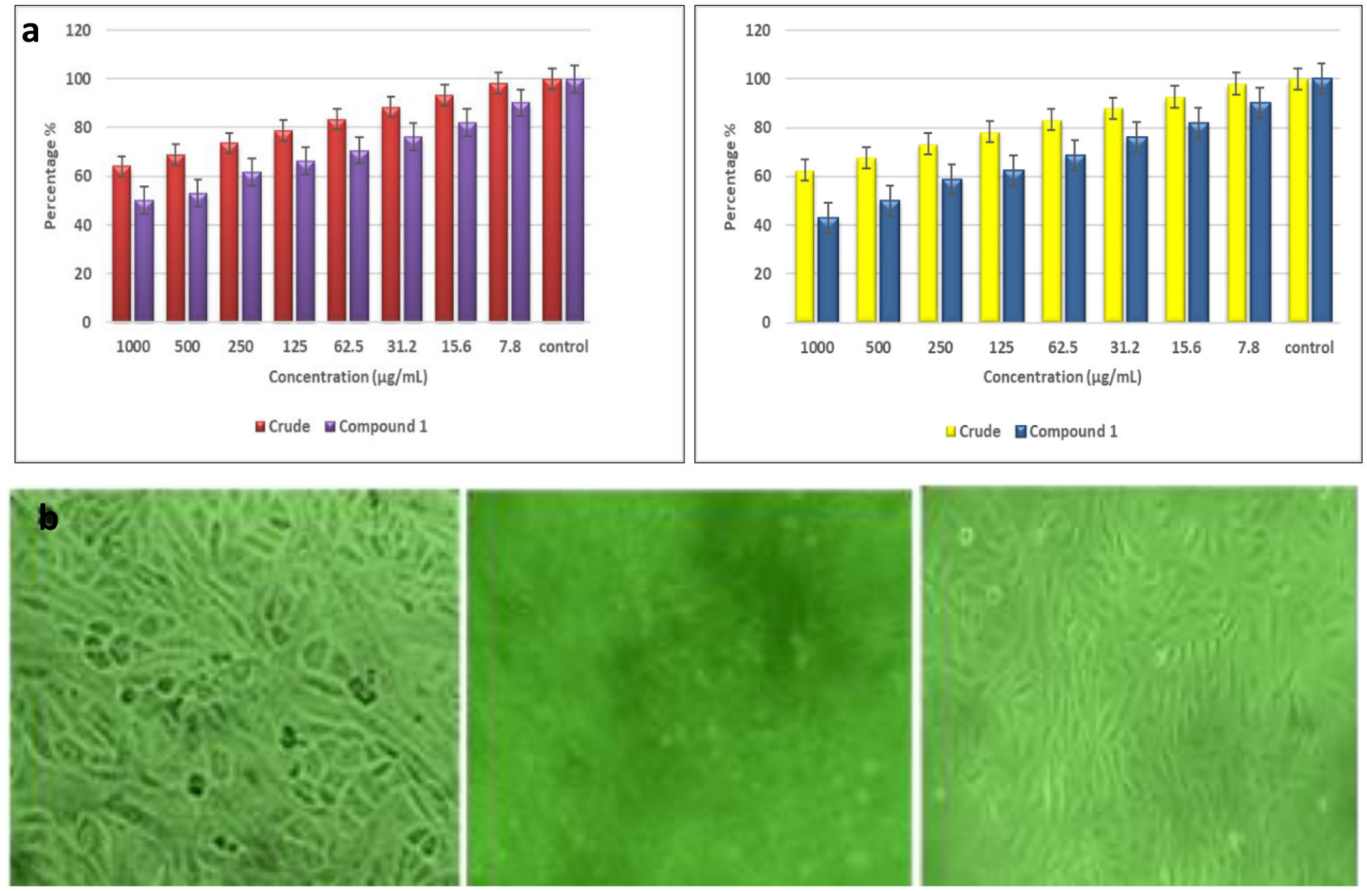
**(a)** Effect of ethyl acetate fungal crude extract and pure compound in the vero cell lines and **(b)** morphologic profiles.

The cytotoxicity effect of the fungal extract and the compounds was conducted on the vero cell line. The results showed that the ethyl acetate extract exhibited notable cytotoxic effects across a range of concentrations, from 7.8 to 1,000 μg/mL, leading to a dose-dependent reduction in cell viability. Specifically, at the highest concentration of 1,000 μL/mL of TPL11 crude extract, cell viability dropped to 64.10%. Whereas, the compounds derived from TPL11 (L11C1) were more active against the cell line, with a decrease in cell viability of 50.12% at a 1,000 μg/mL concentration. The morphological changes observed in the vero cell line following treatment with the ethyl acetate extract and its compound are depicted in Figures 10a, b.

## 4 Discussion

Multidrug-resistant (MDR) microbial infections are anticipated to cause over 10 million deaths annually by 2050. In response to the diminishing efficacy of antibiotics against MDR pathogens, recent research has shifted toward harnessing endophytic fungi as a novel anti-infective strategy. A study reported that the ethyl acetate-derived extracellular metabolites of endophytic *Streptomyces* sp. KCA1, isolated from the leaves of *Phyllanthus niruri*, exhibited antibacterial activity in a disk diffusion assay. The compound 2,4-di-tert-butylphenol demonstrated an MIC of 50 μg/mL against *Escherichia coli* ATCC 25922 and 0.78 μg/mL against *S. aureus* ATCC 29213, whereas the DTB of *F. oxysporum* in the present study displayed 2-fold higher activity with an MIC of 0.39 μg/mL against *S. aureus* ATCC 25923 and MTCC 3160. Furthermore, the IC50 of 2,4-DTBP was determined to be 11.0 μg/mL against the MCF-7 breast cancer cell line and 116.8 μg/mL against the regular vero cell line, whereas our results show more than 50% reduction of vero cells treated with the DTB compound derived from *F. oxysporum* (Seenivasan et al., 2022). Similarly, a newly isolated *Pseudomonas* strain SBMCH11, obtained from soil samples, produced 2,4-di-tert-butylphenol (2,4-DTBP), which exhibited significant antibacterial activity against clinical bacterial isolates, including *Pseudomonas aeruginosa* (12 mm) and *S. aureus* (16 mm). The compound exhibited a lower MIC against *P. aeruginosa* MTCC 741 (31.25 μg/mL) than *S. aureus* MTCC 740 (62.5 μg/mL). The MBC values were determined to be 62.5 μg/mL for *P. aeruginosa* and 125 μg/mL for *S. aureus*. 2,4-DTBP, isolated from thermophilic *Bacillus licheniformis* in an Algerian hot spring, exhibited activity against multidrug-resistant *P. aeruginosa* and *S. aureus* in both pure and mixed cultures, as assessed by a radial diffusion assay at 55°C. 2,4-DTBP extracted from the bulb of *Lilium davidii* var. *willmottiae* and *Fusarium* exhibits a synergistic effect on *Fusarium* wilt in lilies (Velusamy et al., 2024). Similarly, 2,4-di-tert-butylphenol derived from the ethyl acetate extract of *N. sphaerica*, an endophytic fungus associated with the pantropical weed *Euphorbia hirta* L., is responsible for its antioxidant potential (Gautam et al., 2022).

Similarly, the MIC of 2,4-DBP was determined using the microbroth dilution method to assess its inhibitory effect on the growth of *P. aeruginosa*. The MIC was found to be >1,024 μg/mL. Additionally, the growth pattern and cell density of *P. aeruginosa* remained largely unaffected when treated with 2,4-DBP at concentrations of 40, 60, and 80 μg/mL, indicating no impact on the bacterium's growth kinetics. However, a bactericidal assay revealed that 2,4-DBP exhibited synergism with ampicillin in killing *P. aeruginosa* (Mishra et al., 2020). A study reported that 2, 4-di-tert-butylphenol was produced by *Streptomyces* sp. KB1 showed bactericidal anti-MRSA activity against 10 clinical isolates at the concentration level lower than LC_50_ (*p* < 0.05) (Chawawisit et al., 2015). The endophytic fungus *Diaporthe longicolla*, isolated from *Saraca asoca* (Roxb.) Willd., exhibits potent antioxidant activity and significant antibacterial activity against MRSA (Nishad et al., 2021). 2,4-Di-tert-butylphenol, a phenolic compound reported to have antifungal, antimalarial, antioxidant, antibacterial, anti-inflammatory, cytotoxic, antiviral, insecticidal, and nematicidal properties (Nwobodo et al., 2022). 2,4-DTBP isolated from *Dictyota ciliolata* displayed good DPPH Scavenging and anticancer potential against the MCF-7 breast cancer cell line (Aravinth et al., 2023).

At a concentration of 100 μg/mL, 2,4-DTBP significantly inhibited the mycelial growth of *Phytophthora capsici* by approximately 50% compared to the control. It also reported that the compound was used to enhance the efficiency of conventional antibiotics (Zhao et al., 2020).

As a result, 2,4-di-tert-butylphenol (DTB), a secondary metabolite produced by the endophytic fungus *F. oxysporum* isolated from *T. pallida*, provides a foundation for future investigations into its therapeutic potential. Further research is warranted to explore the full spectrum of the bioactive metabolite to elucidate the underlying mechanisms of action. Based on the findings of this study, DTB exhibits promising antibacterial activity and may serve as a potential candidate for the treatment of microbial infections. However, additional comprehensive studies are necessary to evaluate the benefits and potential limitations of DTB across diverse environmental conditions, to optimize its applicability and efficacy in pharmaceutical development.

## 5 Conclusion

The metabolite derived from the fungal extract showcases promise as an effective antibacterial drug capable of addressing a broad spectrum of microbial illnesses. The results of *in silico* and *in vitro* studies suggest that DTB could be implemented in managing infections by resistant human pathogens such as MRSA and VRE. To our knowledge, this is the first report on *F. oxysporum* TPL11 fungus from *T. pallida*. To enhance their application and effectiveness in pharmaceuticals, further research is required to evaluate the full range of costs and benefits associated with deploying fungal endophytes in various environmental settings.

## Data Availability

The datasets presented in this study can be found in online repositories. The names of the repository/repositories and accession number(s) can be found in the article/supplementary material.
